# Hypertriglyceridemia triggered acute pancreatitis in pregnancy – diagnostic approach, management and follow-up care

**DOI:** 10.1186/s12944-019-1180-7

**Published:** 2020-01-04

**Authors:** Gheorghe Cruciat, Georgiana Nemeti, Iulian Goidescu, Stefan Anitan, Andreea Florian

**Affiliations:** 1 Obstetrics and Gynecology, Mother and Child Department, University of Medicine and Pharmacy “Iuliu Hatieganu” Cluj-Napoca, Cluj-Napoca, Romania; 2 Legal Medicine, Community Medicine Department, University of Medicine and Pharmacy “Iuliu Hatieganu” Cluj-Napoca, Cluj-Napoca, Romania

**Keywords:** Hypertriglyceridemia, Pregnancy, Acute pancreatitis

## Abstract

Acute pancreatitis is a pregnancy complication potentially lethal for both the mother and fetus, occurring most frequently in the third trimester or early postpartum. Hypertriglyceridemia may be the cause of important disease in pregnant patients. Patients with triglyceride levels exceeding 1000 mg/dL are at increased risk of developing severe pancreatitis. Diagnostic criteria and management protocols are not specific for pancreatitis complicating pregnancy. Other causes of acute abdominal pain must be considered in the differential diagnosis. Decision-making in the obstetric context is challenging and bears potential legal implications. Pre-pregnancy preventive measures and prenatal antilipemic treatment are mandatory in high risk patients.

## Maintext

Acute pancreatitis (AP) is a rare cause of abdominal pain in pregnancy with a cited incidence of 1/1000–10,000 depending on the diagnostic criteria used and provided accurate diagnosis is not always achieved [1–3]. The majority of cases occur in the third trimester or early postpartum and carry an increased maternal and fetal morbidity and mortality potential [4]. Maternal death rate due to pancreatitis was reportedly 37% and fetal death rate 60%, in decline recently as a result of more advanced diagnostic and therapeutic possibilities [5–7]. Pregnancy associated pancreatitis may occur on the background of gallstone disease, alcohol abuse and hypertriglyceridemia (HTG) [8–10]. It appears that HTG aggravates the severity score and prognosis of disease [7, 11, 12].

AP in pregnancy is a challenge for the clinician since it may be complicated by the onset of labor or obstetrical emergencies. Moreover, the rare occurrence of this condition makes the clinician less suspicious of such an etiology of acute abdominal pain in pregnancy which can delay diagnosis and management and jeopardize fetal outcome. This singularizes further HTG-AP since neither its diagnosis, nor patient approach are fully superposable to the general criteria used for pancreatitis, making this pathology a clinical strategy and medical responsibility issue.

There no currently available obstetric guidelines which highlight the role of HTG and regulate the management of disease are still absent on account of the low incidence rates and scarce clinical data. There are no mentions of pregnancy considerations in guidelines addressing AP management in the general population. Therapy relies on the expertise of the clinician which translates into a susceptibility to medical-legal and malpraxis issues.

### Lipid metabolism in pregnancy and HTG induced pancreatitis pathogenesis

Normal pregnancy is characterized by adaptive lipid metabolism changes meant to ensure placental needs and glucose and lipid requirements of the growing fetus: increased glucose production, progesterone synthesis and lipogenesis, diminished lipolysis [13, 14]. In women with abnormal lipoprotein metabolism these changes lead to severe HTG and may precipitate pancreatitis [15]. Although serum triglycerides (TG) peak in the third trimester, total serum TG level rarely exceeds 300 mg/dL (3.3 mmol/L), a concentration that is not sufficient to cause AP.

HTG may be primary – in congenital chylomicronemia syndrome related to lipoprotein lipase or apoproteins C-II deficiency [16], or secondary in patients with obesity or metabolic syndrome, unrecognized or uncontrolled diabetes mellitus, alcohol consumers, drug use (tamoxifen, steroids, diuretics, beta-blockers, atypical antipsychotics) or may lack any predisposing factor [17–19].

The exact pathogenesis behind HTG-induced AP is not fully understood. TG accumulating around the pancreas are hydrolyzed by pancreatic lipase leading to the accumulation of high levels of free fatty acids [20]. These are believed to be toxic to acinar cells and the capillary endothelium. At the same time, increased chylomicron concentrations, can lead to capillary plugging, ischemia, and acidosis. In this acidic environment, free fatty acids activate trypsinogen and trigger AP [21].

### Diagnostic pitfalls in pregnancy

The diagnosis of AP in pregnancy in difficult since symptoms may mimic any other disease presenting with abdominal pain or the onset of labor. Sometimes, AP onset itself may trigger labor due to peritoneal irritation. Acute medical and surgical conditions presenting with acute abdominal pain must be excluded: myocardial infarction, peptic ulcer, appendicitis, cholecystitis, acute mesenteric ischemia, gastro-intestinal or pancreatic cancer, pyelonephritis etc. Furthermore, obstetric complications must be taken into consideration and carefully ruled out – acute fatty liver of pregnancy, preeclampsia, HELLP syndrome, placental abruption, uterine rupture [22]. The setting of abdominal pain in patients with advanced gestation is particular due to the presence of the enlarged uterus with mechanic upward and lateral displacement on maternal organs, as well as to physiologic changes in pain perception [23]. In term patients with locally complicated disease by acute peri-pancreatic fluid collection it is difficult to achieve diagnosis [16, 17].

HTG-AP cases are typically associated with high serum TG levels (≥1000 mg/dL). However, in certain instances, HTG may be concealed as disease trigger since TG levels drop after a few days of fasting. There have been reports of AP in pregnancy in patients with only HTG as identifiable trigger but at lower levels than the above-mentioned threshold. HTG levels susceptible to activate pancreatitis are thus questionnable [1, 21]. This Moreover, AP diagnosis can be overlooked because serum amylase levels may also be within the normal range at admission [24].

### Management and therapeutic options

The clinical presentation of HTG-AP does not differ from other etiologies, except from the diagnostic traps derived from the obstetric context. Once the correct diagnosis achieved, management principles of pregnant patients follow current guidelines for the general population but are complicated by the decision regarding the timing and route of delivery. It is mandatory that HTG are returned to the normal range [24]. However, there are restrictions regarding chronic antilipemic use in pregnancy due to the lack of consistent studies conducted on humans. Following delivery patient approach returns to non-pregnant population protocols.

*Lipid lowering agents in pregnancy -* Antilipemic agents are the first line of treatment in patients with HTG induced AP of non-pregnant patients. However, these drugs have been only scarcely studied in pregnant women and there is limited information regarding fetal effects [25]. Omega-3 fatty acids are safe for pregnancy use as monotherapy to decrease maternal TG levels, have a rapid onset of action but only moderate effects [26, 27]. Nicotinic acid has only been used in case reports of pregnant women during the first trimester of pregnancy without proven fetal adverse effects [28, 29], however treatment later on in pregnancy has not been advocated. Fibrates use is limited during pregnancy because little data is available from well-designed studies and further research is required to support their use in pregnant women [30]. Statins have potentially teratogenic effects and their use in pregnant patients is restricted, even though studies are controversial [30]. Since moderate risk of fetal developmental abnormality cannot be excluded with statin use, they received FDA (Food and Drug Administration) category X designation [31].

*Insulin and heparin -* Insulin enhances lipoprotein lipase activity and leads to chylomicron degradation thus reducing HTG, while heparin stimulates the releases of lipoprotein lipase which adheres to endothelial cells and decreases serum TG levels [32]. Long-term heparin use will deplete lipoprotein lipase on the surface of endothelial cells having the opposite HTG triggering effect and lead to pancreatitis. Watts et al. describe this in the case report of a pregnant patient. A case report described a pregnant woman who developed HTG-pancreatitis after long-term heparin use [33].

*Apheresis –* therapeutic plasma exchange serves to decrease TG levels, reduce inflammatory cytokines, and replace deficient lipoprotein lipase or apolipoproteins when plasma is used as the replacement fluid [34]. In HTG-AP, apheresis may be used as established by the American Society of Apheresis Guidelines [35]. There are several small-scale studies and clinical case which evaluated the use of plasma exchange in pregnant patients rendering it effective and safe [36–39]. Studies comparing the effect of plasmapheresis versus conservative management on morbidity and mortality in cases of HTG-induced pancreatitis found no statistical difference [40].

### Obstetric decisions and maternal-fetal outcome

#### Pregnancy termination

Interruption of pregnancy is decided by a team involving gastroenterologists, surgeons and obstetricians to minimize fetal loss. Counseling and consent of the couple are mandatory. Confirmed stillbirth, the mandatory use of feto-toxic medication for pancreatitis treatment or organ failure are indications of termination of pregnancy, regardless if requires a term/preterm natural delivery or cesarean section [7]. Pregnancy termination can be regarded as a necessary condition to achieve AP cure [39].

When possible, vaginal delivery is preferable to limit the risk of infection and necrosis associated with laparotomy. However, pregnancy should be terminated by caesarean delivery as soon as possible in case of hyperlipidemic pancreatitis because of the significantly increased risk of maternal and fetal mortality. Emergency surgical delivery is indicated in patients with deteriorated condition after 24–48 h of treatment, no improvement of paralytic ileus, stillbirth, fetal malformations, fetal distress and severe pancreatitis. The potential need to perform prophylactic hysterectomy to avoid the latter risk of uterine wound infection from the pancreatic site should be taken into account, especially in multiparous patients [41].

#### Maternal outcome

Recent data shows that HTG is a risk factor for delayed pancreatic regeneration after AP [42]. Lipid profile must be monitored long term after recovery and the patient must be counseled regarding the risk of recurrent episodes of AP, hyperlipoproteinemia, diabetes melllitus and hyper-viscosity syndrome in later life.

#### Fetal implications

The fetus from an AP pregnancy is exposed to the risk of threatened preterm labor, prematurity and death [7, 43, 44]. Regarding the follow-up of fetuses from hyperlipidemic patients, results of pathology studies performed on 6-month-old deceased fetuses of mothers with hypercholesterolemia compared to mothers with normal cholesterol levels revealed the presence of fatty streaks at aortic level [45, 46]. The hypothesis of fetal metabolic programming suggests that HTG in pregnancy may bear later life consequences on the product of conception. Early adjustments to specific nutritional conditions influence the future physiology and continue to be expressed even in the absence of the condition that initiated them [47]. Fetuses from hypertriglyceridaemic pregnancies are at risk for impaired lipid homeostasis and atherosclerosis through yet poorly understood mechanisms. Studies performed on animals have shown that permanent changes in either DNA (deoxyribonucleic acid) methylation or chromatin modification, or the activation of genes involved in immune pathways and fatty acid metabolism are possible pathways of epigenetic metabolic programming during embryonic/fetal development [48–50].

### Pre-conceptional and prenatal counseling in high risk patients

Patients with HTG taking lipid-lowering therapies should discontinue treatment prior to and during pregnancy since data regarding drug safety are conflicting [29, 41, 51]. They should be informed about the possible exacerbating factors and complications of pregnancy. Lifestyle adjustments are essential, such as a low fat and omega-3 fatty acid rich diet, exercise, abstention from alcohol, and control of secondary triggering factors - diabetes mellitus and drugs which may induce AP (β-adrenal blockers, glucocorticoids, cimetidine, estrogen, oral contraceptives). Excessive weight gain during gestation should be avoided. Glycemic control should be achieved preconceptionally in order to help lower lipid levels, especially in diabetic women [52].

During pregnancy, TG levels should be closely monitored in high risk patients. Timely antilipemic treatment with omega-3 fatty acids and nicotinic acid should de ensured to maintain lipid profile parameters within the normal range. There are authors who support the use of apheresis in the treatment of pregnancy HTG as preventive measure for pancreatitis [53].

The main features which differentiate HTG-AP occurring in pregnancy from the disease occurring in non-pregnant patients are summarized in Table 1. Despite its being an unexpected event in pregnancy, this pathology must be warranted for and looked for given its potentially severe maternal and fetal complications. We may speculate that the rising trend of obesity worldwide may have a negative impact on the incidence of this disease, in pregnancy and otherwise.

**Table 1:** Acute pancreatitis and HTG induced acute pancreatitis features in pregnant versus non-pregnant patients

	Pregnant patient	NON-pregnant patient
Acute pancreatitis		
*Incidence*	1/1000–10000 [3]	10–44/100000 [54]
*Etiology*	Gallstones 65% Alcohol 5–10% *HTG (up to 14.4%)* [9]	Gallstones (40–70%) Alcohol (25–35%) *HTG (2–4%)* [55]
HTG - Acute pancreatitis		
*Pathogenesis*	*Primary* (genetic) & *Secondary* (acquired) disorders of lipoprotein metabolism + Increased lipogenesis & Diminished lipolysis of pregnancy	*Primary* (genetic) & *Secondary* (acquired) disorders of lipoprotein metabolism
*Clinical predictors*	- Unhealthy diet - Metabolic syndrome - Excessive weight gain in pregnancy.	- Unhealthy diet - Metabolic syndrome
*Mortality*	- Maternal (37%), fetal (60%) [56] - 0% maternal and fetal loss rate reported recently but figures are poorer in low income settings	Overall 5–15%, higher for severe disease [57, 58]
*Clinical presentation* *HTG-AP*	- multiparous (75%) [59] - 3rd trimester of pregnancy (50%), early postpartum (38%) - may be complicated by the onset of labor, obstetrical emergencies (placental abruption, eclampsia, HELLP syndrome, uterine rupture)	- generally younger than patients, with other etiologies; - higher chance of systemic inflammatory response syndrome and cardiopulmonary and renal insufficiency;
*Management guidelines*	There are no specific pregnancy related mentions in international guidelines^a^	- 2019 World Society of Emergency Surgery guidelines for the management of severe acute pancreatitis [57] - 2018 Gastroenterological Association Institute Guideline on Initial Management of Acute Pancreatitis [60]
*Severity*	HTG is an independent indicator of poor prognosis in AP. Elevated serum TG independently and proportionally correlate with persistent organ failure in AP patients, regardless of etiology [61].	
*Prophylactic measures*	Lifestyle adjustments Niacin, omega-3 fatty acids Discontinue fibrates/statins Currently there are no guidelines for the management of thepregnant patient at risk for HTG-AP	Lifestyle adjustments Niacin/Fibrates/ Statins
*Initial management*	Fasting, bowel rest Analgesics Hydration & electrolite imbalace correction Measures delayed if diagnostic uncertainty	Fasting, bowel rest Analgesics Hydration & electrolite imbalace correction
*Lipid lowering therapies*	Niacin, omega-3 fatty acids Insulin/heparin infusion Plasmapheresis	Antilipemics Insulin/heparin infusion Plasmapheresis
*Obstetric decison making*	Emergency termination of pregnancy Vaginal delivery preferable	None

^a^ There are no currently available obstetric guidelines which tackle or mention the management of HTG-AP (or AP of other etiologies), nor is there any reference to the obstetric patient in currently available guidelines for the management of AP in the general population

## Conclusions

HTG induced AP is a rare but devastating complication of pregnancy with high mortality potential for both the mother and fetus. Prophylactic measures are essential, namely avoiding excessive weight gain in pregnancy and ensuring lipid and glycemic control prior to and during gestation. Further studies are required to identify safe treatment options during pregnancy. Guidelines are required to establish specific diagnostic criteria or dictate the management in severe cases which mandate challenging obstetric decisions and may trigger medical-legal implications.

## Data Availability

The data in the current paper are publicly available since this a review conducted on the basis of the cited literature.

## Abbreviations

AP: Acute pancreatitis
DNA: Deoxyribonucleic acid
FDA: Food and Drug Administration
HTG: Hypertriglyceridemia
TG: Triglycerides

## References

[1] Zhang DL, Huang Y, Yan L (2013). Thirty-eight cases of acute pancreatitis in pregnancy: a 6-year single center retrospective analysis. J Huazhong Univ Sci Technolog Med Sci.

[2] Bahiyah A, Thanikasalam KP, Lim HC, Ray JR (2015). Severe acute pancreatitis in pregnancy. Case Rep Obstet Gynecol.

[3] Vege SS, Whitcomb DC, Grover S. Clinical manifestations and diagnosis of acute pancreatitis: UpToDate; 2018. https://www.uptodate.com

[4] Terzhumanov R, Uchikov A, Uchikova E, Milchev H, Dimov R, Stefanov C (2004). Acute pancreatitis and pregnancy— analysis of a 10 year period of time. Akush Ginekol.

[5] Sun L, Li W, Geng Y, Shen B, Li J (2011). Acute pancreatitis in pregnancy. Acta Obstet Gynecol Scand.

[6] Papadakis EP, Sarigianni M, Mikhailidis DP, Mamopoulos A, Karagiannis V (2011). Acute pancreatitis in pregnancy: an overview. Eur J Obstet Gynecil Reprod Biol.

[7] Luo L, Zen H, Xu H, Zhu Y, Liu P (2018). Clinical characteristics of acute pancreatitis in pregnancy: experience based on 121 cases. Arch Gynecol Obstet.

[8] Fortson MR, Freedman SN, Webster PD (1995). Clinical assessment of hyperlipidemic pancreatitis. Am J Gastroenterol.

[9] Zhu Y, Pan X, Zeng H (2017). A study on the etiology, severity, and mortality of 3260 patients with acute pancreatitis according to the revised Atlanta classification in Jiangxi, China over an 8-year period. Pancreas.

[10] Cruciat G, Stamatian F, Puscas M, Cruciat C, Ispasoiu F, Muresan D (2007). Acute Pancreatitis in a Pregnant Woman with Acute Fatty Liver Dystrophy. A Case Report. J Gastrointestin Liver Dis.

[11] Kayatas SE, Eser M, Cam C, Cogendez E, Guzin K (2010). Acute pancreatitis associated with hypertriglyceridemia: a life-threatening complication. Arch Gynecol Obstet.

[12] Nawaz H, Koutroumpakis E, Easler J (2015). Elevated serum triglycerides are independently associated with persistent organ failure in acute pancreatitis. Am J Gastroenterol.

[13] Vrijkotte TG, Krukziener N, Hutten BA (2012). Maternal lipid profile during early pregnancy and pregnancy complications and outcomes: the ABCD study. J Clin Endocrinol Metab.

[14] Rauschert S, Gázquez A, Uhl O, Kirchberg FF, Demmelmair H, Ruíz-Palacios M, Prieto-Sánchez MT (2019). Phospholipids in lipoproteins: compositional differences across VLDL, LDL, and HDL in pregnant women. Lipids Health Dis.

[15] Neill AM, Hackett GA, Overton C, Byrne CD (1998). Active management of acute hyperlipidaemic pancreatitis in pregnancy. J Obstet Gynaecol.

[16] Dzenkeviciute Vilma, Skujaite Agne, Rinkuniene Egidija, Petrulioniene Zaneta, Gumbiene Lina, Katkiene Sandra, Laucevicius Aleksandras (2015). Pregnancy-related severe hypertriglyceridemia. Clinical Lipidology.

[17] Adiamah A, Psaltis E, Crook M, Lobo DN (2017). A systematic review of the epidemiology, pathophysiology and current management of hyperlipidaemic pancreatitis. Clin Nutr.

[18] Cahalane AM, Smith MJ, Ryan J, Maguire D (2012). Acute pancreatitis secondary to gestational hypertriglyceridaemia. Case Rep Med.

[19] Procopciuc LM, Hazi GM, Caracostea G, Nemeti G, Olteanu I, Stamatian F (2011). Apolipoprotein E polymorphism - a risk factor in Romanian pregnant women with preeclampsia. GINECO RO volume: 7. Issue.

[20] Kadikoylu G, Yukselen V, Yavasoglu I, Coskun A, Karaoglu AO, Bolaman Z (2010). Emergent therapy with thera- peutic plasma exchange in acute recurrent pancreatitis due to severe hypertriglyceridemia. Transfusion and Apheresis Sci- ence.

[21] Lindkvist B, Appelros S, Regnér S, Manjer J (2012). A prospective cohort study on risk of acute pancreatitis related to serum triglycerides, cholesterol and fasting glucose. Pancreatology.

[22] Woodhead N, Nkwam O, Caddick V, Morad S, Mylvaganam S (2019). Surgical causes of acute abdominal pain in pregnancy. Obstet Gynaecol.

[23] Zachariah SK, Fenn M, Jacob K, Arthungal SA, Zachariah SA (2019). Management of acute abdomen in pregnancy: current perspectives. Int J Women's Health.

[24] Sezgin O, Özdoğan O, Yaraş S, Üçbilek E, Altıntaş E (2019). Evaluation of hypertriglyceridemia-induced acute pancreatitis: a single tertiary care unit experience from Turkey. Turk J Gastroenterol.

[25] Briggs G (2016). Examining the safety of lipid-lowering drugs in pregnancy.

[26] Barrett HL, Marloes DN, McIntyre HD, Callaway LK (2014). Normalizing metabolism in diabetic pregnancy: is it time to target lipids?. Diabetes Care.

[27] Stone NJ, Robinson JG, Lichtenstein AH (2014). 2013 ACC/AHA cholesterol guideline panel. 2013 ACC/AHA guideline on the treatment of blood cholesterol to reduce atherosclerotic cardiovascular risk in adults: a report of the American College of Cardiology/American Heart Association task force on practice guidelines. Circulation.

[28] Al-Shali K, Wang J, Fellows F (2002). Successful pregnancy out- come in a patient with severe chylomicronemia due to compound heterozygosity for mutant lipoprotein lipase. Clin Biochem.

[29] Tsai EC, Brown JA, Veldee MY (2004). Potential of essential fatty acid deficiency with extremely low fat diet in lipoprotein lipase deficiency during pregnancy: a case report. BMC Pregnancy Childbirth.

[30] Rawla P, Sunkara T, Thandra KC, Gaduputi V (2018). Hypertriglyceridemia-induced pancreatitis: updated review of current treatment and preventive strategies. Clin J Gastroenterol.

[31] Newman CB, Preiss D, Tobert JA, Jacobson TA, Page RL (2019). Statin safety and associated adverse events: a scientific statement from the American Heart Association. Arterioscler Thromb Vasc Biol.

[32] Korn ED (1955). Clearing factor, a heparin-activated lipoprotein lipase. II. Substrate specificity and activation of coconut oil. J Biol Chem.

[33] Watts GF, Cameron J, Henderson A, Richmond W (1991). Lipoprotein lipase deficiency due to long-term heparinization presenting as severe hypertriglyceridaemia in pregnancy. Postgrad Med J.

[34] Yeh JH, Chen JH, Chiu HC (2003). Plasmapheresis for hyperlipidemic pancreatitis. J Clin Apher.

[35] Schwartz J, Padmanabhan A, Aqui N, Balogun RA, Connelly-Smith L (2016). Guidelines on the use of therapeutic apheresis in clinical practice-evidence-based approach from the writing Committee of the American Society for apheresis: the seventh special issue. J Clin Apher.

[36] Klingel R, Go Hlen B, Schwarting A, Himmelsbach F, Straube R (2003). Differential indication of lipoprotein apheresis during pregnancy. Ther Apher Dial.

[37] Safi F, Toumeh A, Abuissa Qadan MA, Karaz R, AlAkdar B, Assaly R (2014). Management of familial hypertriglyceridemia-induced pancreatitis during pregnancy with therapeutic plasma exchange: a case report and review of literature. Am J Ther.

[38] Eren Gulay, Hergünsel Oya, Altun Dilek, Cukurova Zafer, Yasar Levent (2012). An alternative treatment in hypertriglyceridemia-induced acute pancreatitis in pregnancy: Plasmapheresis. Journal of Anaesthesiology Clinical Pharmacology.

[39] Huang C, Liu J, Lu Y, Fan J, Wang X, Liu J, Zhang W, Zeng Y (2016). Clinical features and treatment of hypertriglyceridemia-induced acute pancreatitis during pregnancy: a retrospective study. J Clin Apher.

[40] Chen JH, Yeh JH, Lai HW, Liao CS (2004). Therapeutic plasma exchange in patients with hyperlipidemic pancreatitis. World J Gastroenterol.

[41] Goldberg AS, Hegele RA (2012). Severe hypertriglyceridemia in preg- nancy. J Clin Endocrinol Metab.

[42] Yang N, Li B, Pan Y, Tu J, Liu G (2019). Hypertriglyceridaemia delays pancreatic regeneration after acute pancreatitis in mice and patients. Gut.

[43] Tang M, Xu JM, Song SS, Mei Q, Zhang LJ (2018). What may cause fetus loss from acute pancreatitis in pregnancy: analysis of 54 cases. Medicine (Baltimore).

[44] Cortés-Vásquez J, Noreña I, Mockus I (2018). Hypertriglyceridemia and adverse outcomes during pregnancy. Rev Fac Med.

[45] Napoli C, D'Armiento FP, Mancini FP (1997). Fatty streak formation occurs in human fetal aortas and is greatly enhanced by maternal hypercholesterolemia. Intimal accumulation of low density lipoprotein and its oxidation precede monocyte recruitment into early atherosclerotic lesions. J Clin Invest.

[46] Frantz E, Menezes HS, Lange KC (2012). The effect of maternal hypercholesterolemia on the placenta and fetal arteries in rabbits. Acta Cir Bras.

[47] Grimes SB, Wild R. Effect of pregnancy on lipid metabolism and lipoprotein levels: Endotext. https://www.ncbi.nlm.nih.gov/books/NBK498654/29714937

[48] DeRuiter MC, Alkemade FE, Gittenberger-de Groot AC, Poelmann RE, Havekes LM, van Dijk KW (2008). Maternal transmission of risk for atherosclerosis. Curr Opin Lipidol.

[49] Goharkhay N, Tamayo EH, Yin H (2008). Maternal hypercholesterolemia leads to activation of endogenous cholesterol synthesis in the offspring. Am J Obstet Gynecol.

[50] Palinski W, D'Armiento FP, Witztum JL (2001). Maternal hypercholesterolemia and treatment during pregnancy influence the long-term progression of atherosclerosis in offspring of rabbits. Circ Res.

[51] Zarek J, Koren G (2014). The fetal safety of statins: a systematic review and meta-analysis. J Obstet Gynaecol Can.

[52] MacIntosh MCM, Fleming KM, Bailey JA (2006). Perinatal mortal- ity and congenital anomalies in babies of women with type 1 or type 2 diabetes in England, Wales and Northern Ireland: popula- tion based study. BMJ.

[53] Basar R, Uzum AK, Canbaz B, Dogansen SC, Kalayoglu-Besisik S (2013). Therapeutic apheresis for severe hypertriglyceridemia in pregnancy. Arch Gynecol Obstet.

[54] Spanier BW, Dijkgraaf MG, Bruno MJ (2008). Epidemiology, aetiology and outcome of acute and chronic pancreatitis: an update. Best Pract Res Clin Gastroenterol.

[55] Valdivielso P, Ramirez-Bueno A, Ewald N (2014). Current knowledge of hypertriglyceridemic pancreatitis. Eur J Intern Med.

[56] Hernandez A, Petrov MS, Brooks DC, Banks PA, Ashley SW, Tavakkolizadeh A (2007). Acute pancreatitis and pregnancy: a 10-year single center experience. J Gastrointest Surg.

[57] Leppäniemi A, Tolonen M, Tarasconi A (2019). 2019 WSES guidelines for the management of severe acute pancreatitis. World J Emerg Surg.

[58] Koutroumpakis E, Slivka A, Furlan A, Dasyam AK, Dudekula A (2017). Management and outcomes of acute pancreatitis patients over the last decade: A US tertiary-center experience. Pancreatology.

[59] Ducarme G, Maire F, Chatel P (2014). Acute pancreatitis during pregnancy: a review. J Perinatol.

[60] Crockett SD (2018). American Gastroenterological Association Institute guideline on initial management of acute pancreatitis. Gastroenterology.

[61] Gayam Vijay, Mandal Amrendra Kumar, Garlapati Pavani, Khalid Mazin, Gill Arshpal, Mowyad Khalid (2018). Moderate Hypertriglyceridemia Causing Recurrent Pancreatitis: A Case Report and the Literature Review. Case Reports in Gastrointestinal Medicine.

